# An effective tool for predicting survival in breast cancer patients with *de novo* lung metastasis: Nomograms constructed based on SEER

**DOI:** 10.3389/fsurg.2022.939132

**Published:** 2023-01-06

**Authors:** WenYi Wang, JiaJing Liu, YuQiu Chen, XiaoFan Xu, LiQun Huo, XuLin Wang, Jun Gu

**Affiliations:** ^1^ Research Institute of General Surgery, Affiliated Jinling Hospital, Medical School, Nanjing University, Nanjing, China; ^2^ Department of General Surgery, Jinling Hospital, Nanjing Medical University, Nanjing, China

**Keywords:** breast cancer lung metastasis, nomogram, prognosis, SEER, survival

## Abstract

**Background & objectives:**

An effective tool for forecasting the survival of BCLM is lacking. This study aims to construct nomograms to predict overall survival (OS) and breast cancer-specific survival (BCSS) in breast cancer patients with *de novo* lung metastasis, and to help clinicians develop appropriate treatment regimens for breast cancer lung metastasis (BCLM) individuals.

**Methods:**

We gathered clinical data of 2,537 patients with BCLM between 2010 and 2015 from the Surveillance, Epidemiology, and End Results (SEER) database. Cox regression analysis was employed to identify independent prognostic parameters for BCLM, which were integrated to establish nomograms by R software. The discriminative ability and predictive accuracy of the nomograms were assessed using the concordance index (C-index), receiver operating characteristic (ROC) curves, and calibration plots. Kaplan–Meier analyses were applied to evaluate the clinical utility of the risk stratification system and investigate the survival benefit of primary site surgery, chemotherapy, and radiotherapy for BCLM patients.

**Results:**

Two nomograms shared common prognostic indicators including age, marital status, race, laterality, grade, AJCC T stage, subtype, bone metastasis, brain metastasis, liver metastasis, surgery, and chemotherapy. The results of the C-index, ROC curves, and calibration curves demonstrated that the nomograms exhibited an outstanding performance in predicting the prognosis of BCLM patients. Significant differences in the Kaplan–Meier curves of various risk groups corroborated the nomograms' excellent stratification. Primary site surgery and chemotherapy remarkably improved OS and BCSS of BCLM patients whether the patients were at low-risk or high-risk, but radiotherapy did not.

**Conclusions:**

We successfully developed prognostic stratification nomograms to forecast prognosis in BCLM patients, which provide important information for indicating prognosis and facilitating individualized treatment regimens for BCLM patients.

## Introduction

The incidence of breast cancer (BC) is highest among malignant tumors, and breast cancer is one of the leading causes of cancer-related death worldwide (1). When breast cancer patients are first diagnosed, approximately 5%–10% of them have distant metastasis (2). The lung is the second most common metastatic site in breast cancer patients (3). In a study encompassing 11,568 patients with metastatic breast cancer, 36.4% of patients had lung metastasis (4). Despite amelioration in diverse treatments, including radiotherapy, chemotherapy, or targeted therapy, the prognosis of breast cancer patients with lung metastasis remains poor with a median survival of 13 to 21 months (4, 5). In addition, a large proportion of breast cancer lung metastasis (BCLM) patients always suffer severe complications synchronously, leading to a high mortality rate in BCLM patients. The survival-related risk factors of BCLM have been reported (4), but an effective tool for forecasting the survival of BCLM is lacking.

Recently, nomograms have been extensively used in tumor prediction as a reliable predicted tool (6, 7). Thus, in this study, we exploited data from the Surveillance, Epidemiology, and End Results (SEER) database to identify independent prognostic factors associated with survival in BCLM patients, and developed nomograms to predict OS and BCSS in patients with BCLM. Besides, we built a risk stratification system based on the nomogram models and evaluated the benefit of different treatments in diverse stratified risk groups.

## Methods

### Data collection and study design

**Figure 1:**
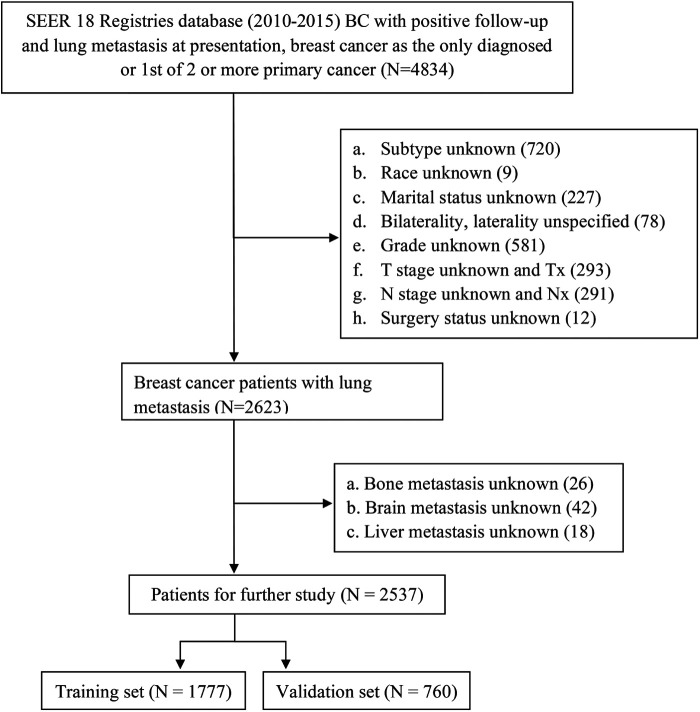
The flowchart of patients selected in the present study.

We used SEER*Stat 8.3.9 to acquire the data of adult patients who were primarily diagnosed with breast cancer lung metastasis between 2010 and 2015 (*n* = 4,834). Patient demographic characteristics (sex, age, marital status, and race), disease characteristics (site, laterality, grade, American Joint Committee on Cancer (AJCC) T stage, AJCC N stage, molecular type, and distant metastatic sites), treatment modalities (surgery, chemotherapy, and radiotherapy) and survival status (survival time, vital status and cause of death) were included in our study. The selection process of detailed inclusion and exclusion criteria is displayed in Figure 1. Eventually, 2,537 eligible patients were extracted for further study. There was no need for formal consent in this type of retrospective study.

### Statistical analysis

We randomly allocated eligible patients into training and validation cohorts at a ratio of 7:3. According to the cause of death classification in the SEER database, the time from the date of diagnosis to death from any cause was defined as overall survival (OS), and the time from the date of diagnosis to the date of death from breast cancer was defined as breast cancer-specific survival (BCSS).

The characteristics of the training cohort and the validation cohort were compared using the chi-squared test. Univariate and multivariate Cox analyses were utilized to identify independent risk factors for prognosis. All of the identified independent risk factors were employed to construct nomograms for estimating 1-, 2-, and 3-year OS and BCSS. The discriminative ability of the nomograms was assessed using the C-index and ROC curves. The predictive capacity of the nomograms was tested by calibration plots, which can estimate the predicted and observed survival probability. Based on the aggregate score of the clinicopathological baseline data in the nomograms, breast cancer patients with lung metastasis were divided into low-risk and high-risk groups. Kaplan–Meier survival analyses were applied to assess the discriminatory power of the risk stratification system and investigate the survival benefit of primary site surgery, chemotherapy, and radiotherapy for BCLM patients in different risk groups.

All of these analyses were executed using packages (including caret, rms, foreign, survival, and survivalROC) in R software (version 4.0.4; http://www.r-project.org). A two-sided *P* < 0.05 was considered statistically significant.

## Results

### Patient characteristics

Through rigorous selection, as shown in Figure 1, a total of 2,537 breast cancer patients with initial lung metastasis were included for analysis (1,777 patients in the training set and 760 patients in the validation set). Patients of the entire cohort were found to have a median survival time of 25 months (95% CI: 24–27), and have 0.695 (95% CI: 0.677–0.713), 0.509 (95% CI: 0.490–0.529), 0.388 (95% CI 0.369–0.407) of 1-, 2-, 3- year survival rates respectively. Demographics and clinicopathologic characteristics of BCLM patients were displayed in Table 1. In the training cohort, most of the patients were female (98.4%, 1748) and white (72.8%, 1294), and the age of patients was mainly distributed among middle-aged and senior people (40–59 years old: 38.4%, 682; 60–79 years old: 44.1%, 784). BCLM patients with higher grades and higher T stages accounted for a higher proportion. Moreover, more than half of BCLM patients were HR+/HER2-. Furthermore, the proportion of chemotherapy-received patients was almost two times of the surgery- or radiotherapy-received, 65.5%, 32.6%, and 30.0% in the training cohort, respectively. In addition, the incidence of bone metastasis in BCLM patients was the highest (53.0%), followed by liver metastasis (28.1%).

**Table 1:** Demographics and clinicopathologic characteristics of the cohort with BCLM.

Variables	Overall (*N* = 2537)	Training cohort (*N* = 1777)	Validation cohort (*N* = 760)	*P*-value
**Sex**				0.862
Female	2,497 (98.4%)	1,748 (98.4%)	749 (98.6%)	
Male	40 (1.6%)	29 (1.6%)	11 (1.4%)	
**Age**				0.708
<40	166 (6.5%)	113 (6.4%)	53 (7.0%)	
40–59	956 (37.7%)	682 (38.4%)	274 (36.1%)	
60–79	1,130 (44.5%)	784 (44.1%)	346 (45.5%)	
80+	285 (11.2%)	198 (11.1%)	87 (11.4%)	
**Marital status**				0.573
Married	1,116 (44.0%)	788 (44.3%)	328 (43.2%)	
Unmarried	1,421 (56.0%)	989 (55.7%)	432 (56.8%)	
**Race**				
White	1,833 (72.3%)	1,294 (72.8%)	539 (70.9%)	0.6
Black	484 (19.1%)	334 (18.8%)	150 (19.7%)	
Other	220 (8.7%)	149 (8.4%)	71 (9.3%)	
**Site**				0.676
Inner	277 (10.9%)	200 (11.3%)	77 (10.1%)	
Outer	733 (28.9%)	515 (29.0%)	218 (28.7%)	
Other	1,527 (60.2%)	1,062 (59.8%)	465 (61.2%)	
**Laterality**				0.516
Left	1,272 (50.1%)	883 (49.7%)	389 (51.2%)	
Right	1,265 (49.9%)	894 (50.3%)	371 (48.8%)	
**Grade**				0.511
I-II	1,076 (42.4%)	746 (42.0%)	330 (43.4%)	
III-IV	1,461 (57.6%)	1,031 (58.0%)	430 (56.6%)	
**AJCC_T**				0.655
T1-2	957 (37.7%)	665 (37.4%)	292 (38.4%)	
T3-4	1,580 (62.3%)	1,112 (62.6%)	468 (61.6%)	
**AJCC_N**				0.789
N0	523 (20.6%)	369 (20.8%)	154 (20.3%)	
N1-3	2,014 (79.4%)	1,408 (79.2%)	606 (79.7%)	
**Subtype**				0.248
HR+/HER2−	1,293 (51.0%)	897 (50.5%)	396 (52.1%)	
HR+/HER2+	471 (18.6%)	346 (19.5%)	125 (16.4%)	
HR−/HER2+	276 (10.9%)	185 (10.4%)	91 (12.0%)	
HR−/HER2−	497 (19.6%)	349 (19.6%)	148 (19.5%)	
**Bone**				0.165
No	1,215 (47.9%)	835 (47.0%)	380 (50.0%)	
Yes	1,322 (52.1%)	942 (53.0%)	380 (50.0%)	
**Brain**				0.199
No	2,307 (90.9%)	1,607 (90.4%)	700 (92.1%)	
Yes	230 (9.1%)	170 (9.6%)	60 (7.9%)	
**Liver**				0.244
No	1,842 (72.6%)	1,278 (71.9%)	564 (74.2%)	
Yes	695 (27.4%)	499 (28.1%)	196 (25.8%)	
**Surgery**				0.378
No	1,723 (67.9%)	1,197 (67.4%)	526 (69.2%)	
Yes	814 (32.1%)	580 (32.6%)	234 (30.8%)	
**Chemotherapy**				0.174
No/Unknown	897 (35.4%)	613 (34.5%)	284 (37.4%)	
Yes	1,640 (64.6%)	1,164 (65.5%)	476 (62.6%)	
**Radiation**				0.371
No/Unknown	1,762 (69.5%)	1,244 (70.0%)	518 (68.2%)	
Yes	775 (30.5%)	533 (30.0%)	242 (31.8%)	

For marital status, unmarried consists of single, divorced, separated, and widowed; For race, ‘other’ includes American Indian, AK Native, Asian, and Pacific Islander; For grade, Grade I means well-differentiated, grade II means moderately differentiated, grade III means poorly differentiated, Grade IV means undifferentiated or anaplastic.

### Univariate and multivariate cox regression analysis

The results generated by univariate Cox analysis are listed in Supplementary Table S1. We identified twelve variables including age, marital status, race laterality, grade, AJCC T stage, subtype, bone metastasis, brain metastasis, liver metastasis, surgery, and chemotherapy, that were statistically associated with the OS and BCSS of BCLM patients. These twelve variables were included in multivariate analysis, and the results suggested that all of the twelve variables were confirmed as final prognostic factors for OS and BCSS (Table 2).

**Table 2:** Multivariate Cox regression analysis for overall survival (OS) and breast cancer-specific survival (BCSS) of BCLM patients in the training cohort.

Variables	OS			BCSS		
	HR (95% CI)	*P*-value	Points	HR (95% CI)	*P*-value	Points
**Sex**						
Female	–	–	–	–	–	–
Male	–	–	–	–	–	–
**Age**						
<40	Reference		0	Reference		0
40–59	1.288 (1.005–1.651)	0.0453	19	1.322 (1.019–1.715)	0.0358	20
60–79	1.480 (1.155–1.897)	0.0020	30	1.427 (1.099–1.854)	0.0077	26
80+	2.458 (1.844–3.277)	0.0000	68	2.264 (1.666–3.078)	0.0000	60
**Marital status**						
Married	Reference		0	Reference		0
Unmarried	1.319 (1.177–1.478)	0.0000	21	1.2991.151–1.466)	0.0000	19
**Race**						
White	Reference		14	Reference		15
Black	1.228 (1.065–1.415)	0.0047	30	1.184 (1.017–1.377)	0.0293	27
Other	0.833 (0.678–1.023)	0.0819	0	0.814 (0.653–1.014)	0.0660	0
**Site**						
Inner	–	–	–	–	–	–
Outer	–	–	–	–	–	–
Other	–	–	–	–	–	–
**Laterality**						
Left	Reference		0	Reference		0
Right	1.160 (1.041–1.293)	0.0074	11	1.163 (1.036–1.306)	0.0104	11
**Grade**						
I-II	Reference		0	Reference		0
III-IV	1.402 (1.236–1.590)	0.0000	26	1.493 (1.304–1.709)	0.0000	20
**AJCC_T**						
T1-2	Reference		0	Reference		0
T3-4	1.307 (1.164–1.468)	0.0000	20	1.354 (1.196–1.534)	0.0000	22
**AJCC_N**						
N0	–	–	–	–	–	–
N1-3	–	–	–	–	–	–
**Subtype**						
HR+/HER2−	Reference		18	Reference		18
HR+/HER2+	0.789 (0.669–0.930)	0.0048	0	0.777 (0.652–0.926)	0.0047	0
HR−/HER2+	1.207 (0.982–1.485)	0.0738	32	1.102 (0.881–1.377)	0.3950	26
HR−/HER2−	2.937 (2.492–3.462)	0.0000	100	3.051 (2.566–3.629)	0.0000	100
**Bone**						
No	Reference		0	Reference		0
Yes	1.355 (1.198–1.532)	0.0000	23	1.3206 (1.1583–1.5057)	0.0000	20
**Brain**						
No	Reference		0	Reference		0
Yes	1.926 (1.618–2.294)	0.0000	50	1.9160 (1.593–2.305)	0.0000	48
**Liver**						
No	Reference		0	Reference		0
Yes	1.644 (1.451–1.863)	0.0000	38	1.778 (1.559–2.028)	0.0000	42
**Surgery**						
No	Reference		20	Reference		21
Yes	0.770 (0.680–0.872)	0.0000	0	0.747 (0.654–0.853)	0.0000	0
**Chemotherapy**						
No/Unknown	Reference		35	Reference		32
Yes	0.633 (0.556–0.721)	0.0000	0	0.648 (0.563–0.745)	0.0000	0
**Radiation**						
No/Unknown	–	–	–	–	–	–
Yes	–	–	–	–	–	–

### Construction and validation of nomograms

**Figure 2:**
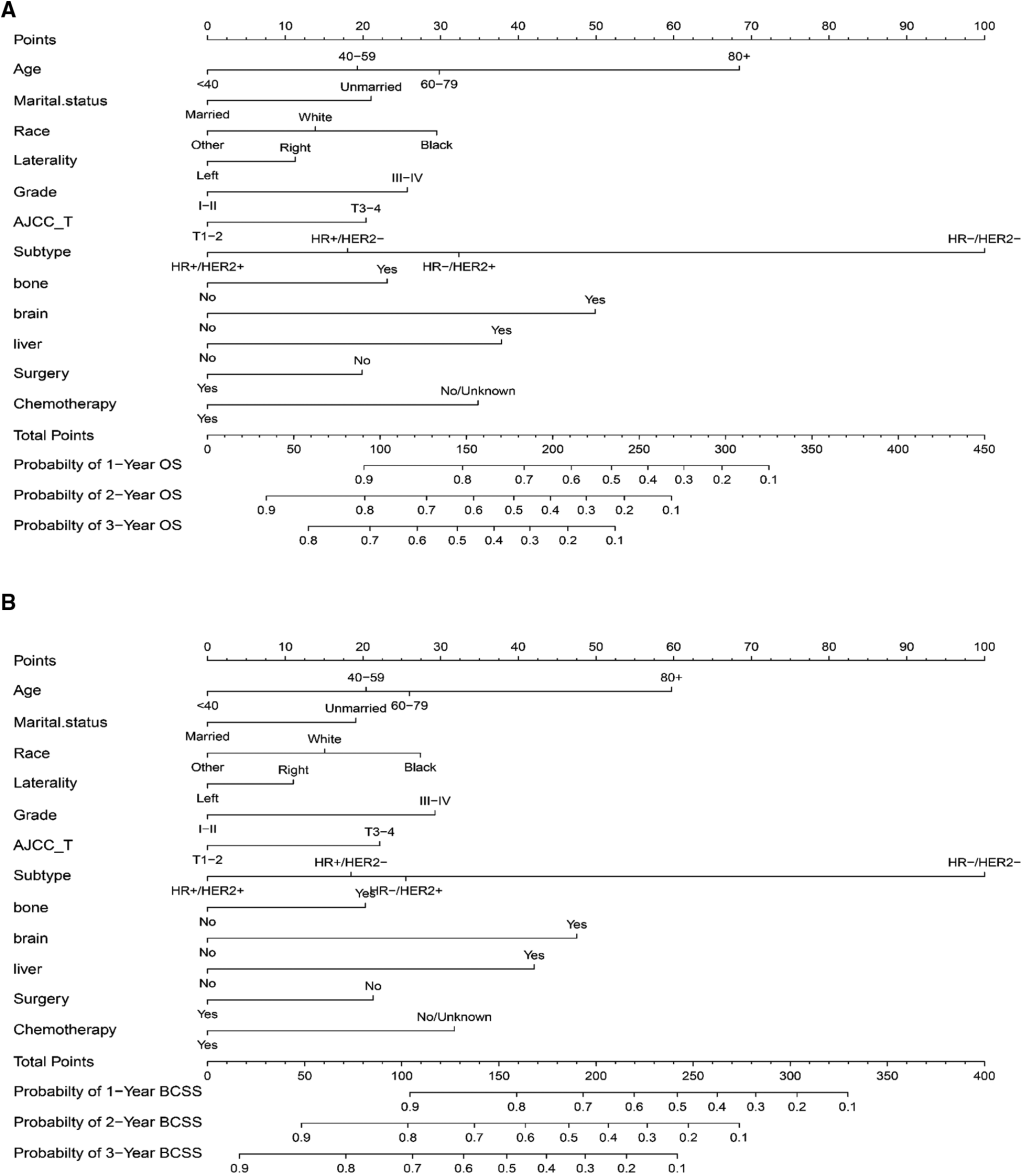
Nomograms for predicting 1-, 2-, and 3-year **(A)** overall survival (OS) and **(B)** breast cancer-specific survival (BCSS) for BCLM patients. HR, hormone receptor; HER2, Human epidermal growth factor receptor 2; AJCC, American Joint Committee on Cancer.

The twelve final prognostic factors were then used in the nomogram establishment. In each nomogram, every variable was assigned a special score according to the point scale (Table 2). The nomogram showed that the tumor subtype contributed the most to prognosis, followed by age and brain metastasis. By calculating the sum scores of each patient's clinical covariates, we can estimate the 1-year, 2-year, and 3-year OS and BCSS on the “total points” axis (Figures 2A,B).

**Figure 3:**
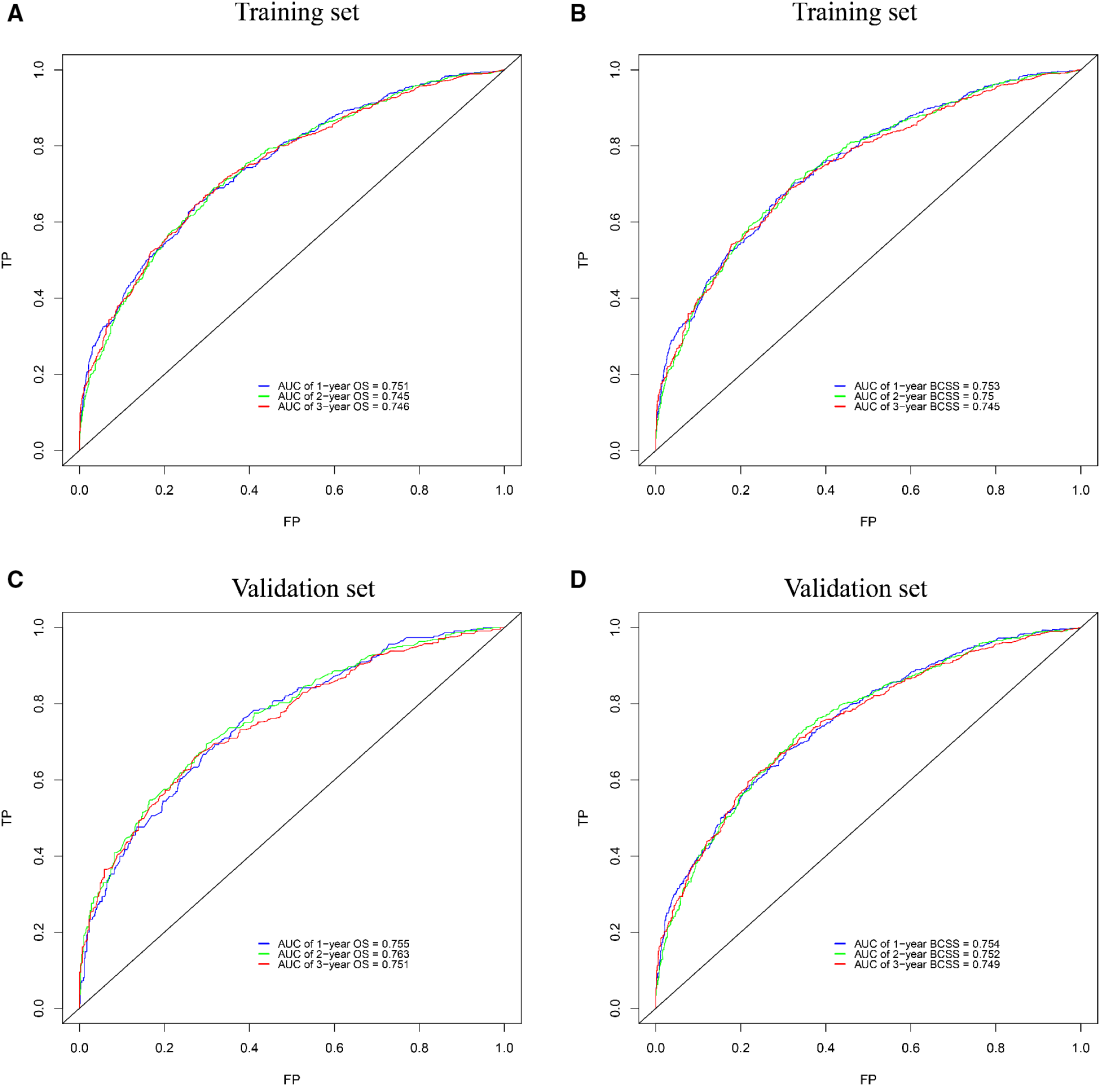
ROC curves for survival prediction of BCLM patients. **(A,B)** ROC curves of 1-, 2-, and 3-year overall survival (OS) and breast cancer-specific survival (BCSS) in the training set; **(C,D)** ROC curves of 1-, 2-, and 3-year OS and BCSS in the validation set. ROC: receiver operating characteristic, AUC: the area under the time-dependent ROC curve.

**Figure 4:**
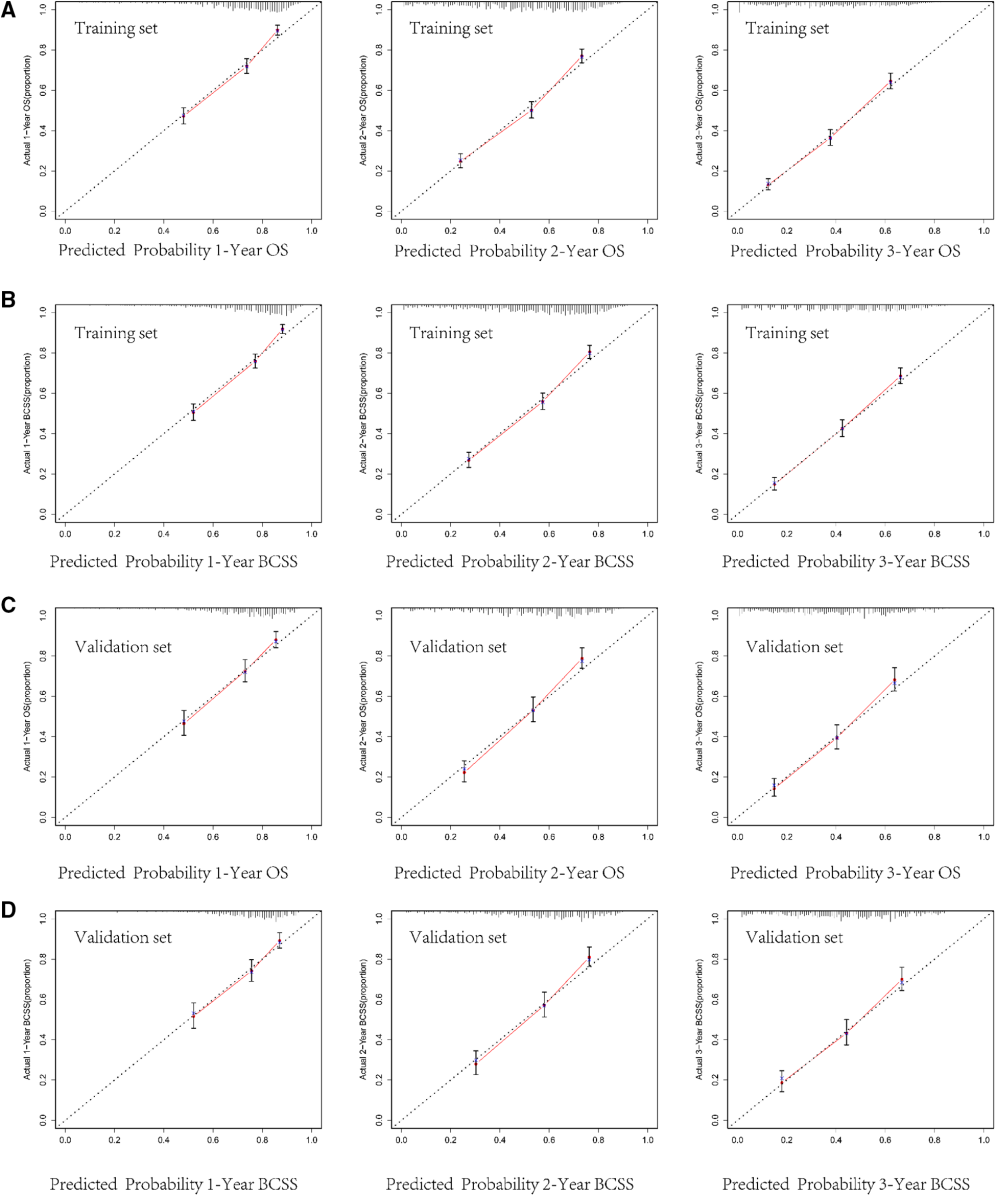
Calibration curves for predicting 1-, 2-, and 3-year overall survival (OS) and breast cancer-specific survival (BCSS) in the training cohort **(A,B)** and in the validation cohort **(C,D).**

The internal verification of the training set and external verification of the validation set were used to assess the credibility of the nomograms. The C-index of the OS nomogram was 0.701 in the training cohort and 0.699 in the validation cohort, and the C-index of the BCSS nomogram was 0.708 in the training group and 0.697 in the validation group (Supplementary Table S2). In the training set, the area under the time-dependent ROC curve (AUC) of the nomogram to predict 1-, 2- and 3-year OS and BCSS ranged from 0.745 to 0.753 (Figures 3A,B). In the validation cohort, the AUC values of the nomogram to predict 1-, 2- and 3-year OS and BCSS ranged from 0.749 to 0.763 (Figures 3C,D). The calibration curves in both the training cohort and validation cohort showed good consistency between the model-based predictions and the actual observations (Figure 4).

### Risk stratification system

**Figure 5:**
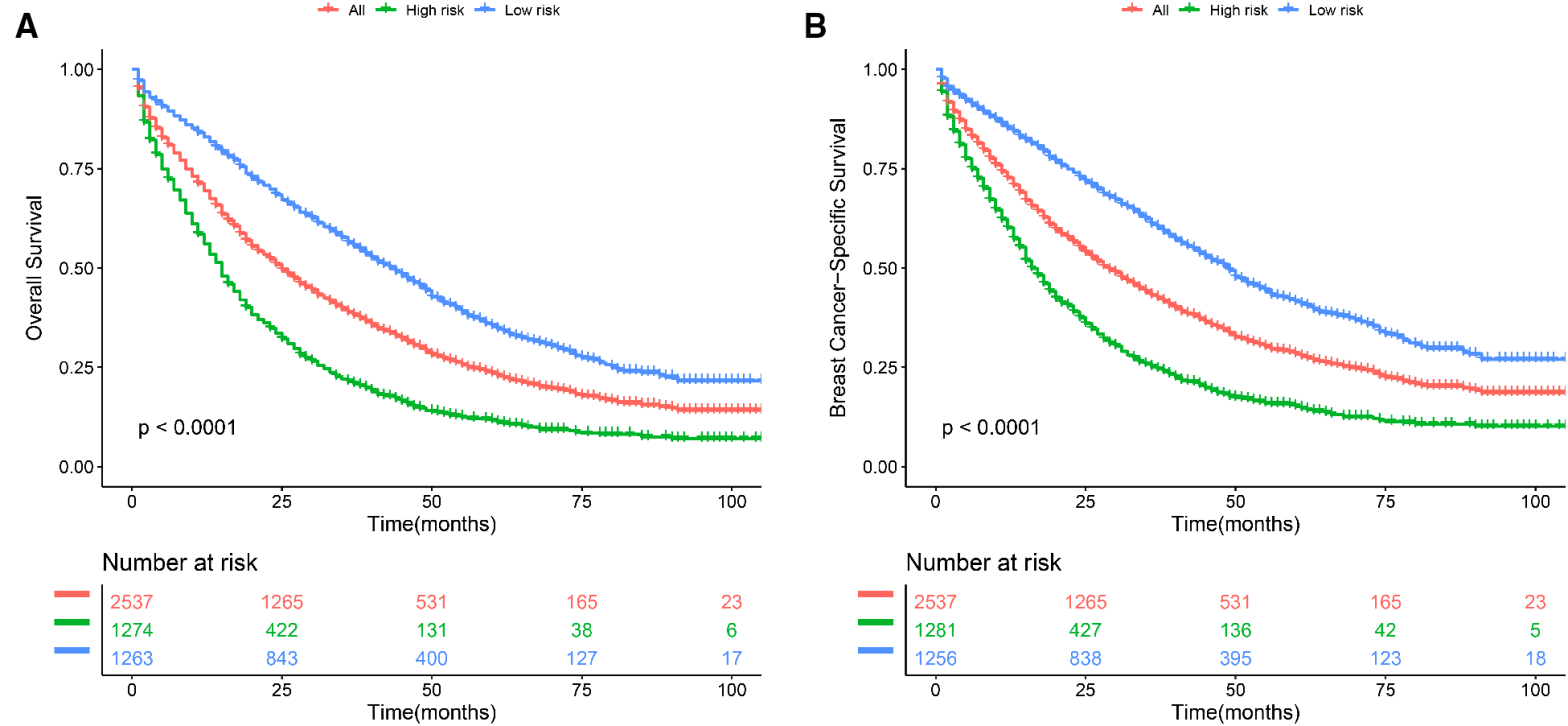
Kaplan–Meier curve of (**A)** overall survival (OS) and **(B)** breast cancer-specific survival (BCSS) to test the stratification system in the total cohort. All: all BCLM patients.

Based on the nomogram, we further established a risk classification and evaluated the impact of clinicopathological baseline data risk on the prognosis of patients. We calculated the sum scores of ten independent predictors (including age, marital status, race, laterality, grade, AJCC T stage, subtype, bone metastasis, brain metastasis, and liver metastasis), only demographic characteristics and disease characteristics were included. The median of the sum scores was set as the threshold. Above the median of predicted total scores was defined as high risk, as well below the median of predicted total scores was defined as low risk. For OS, BCLM patients were split into the low-risk group (scores < 147) and the high-risk group (scores ≥147). For BCSS, BCLM patients were separated into the low-risk group (scores < 139) and the high-risk group (scores ≥139). In the total cohort, the patients at low risk had better OS and BCSS compared with all BCLM patients, the BCLM patients at high risk showed worse OS and BCSS compared with all BCLM patients (Figures 5A,B). The Kaplan-Meier curves visibly differentiated the prognostic differences between the low-risk and the high-risk groups, indicating the excellent clinical utility of the nomograms.

### Kaplan Meier analyses of different treatments in stratified risk groups

**Figure 6:**
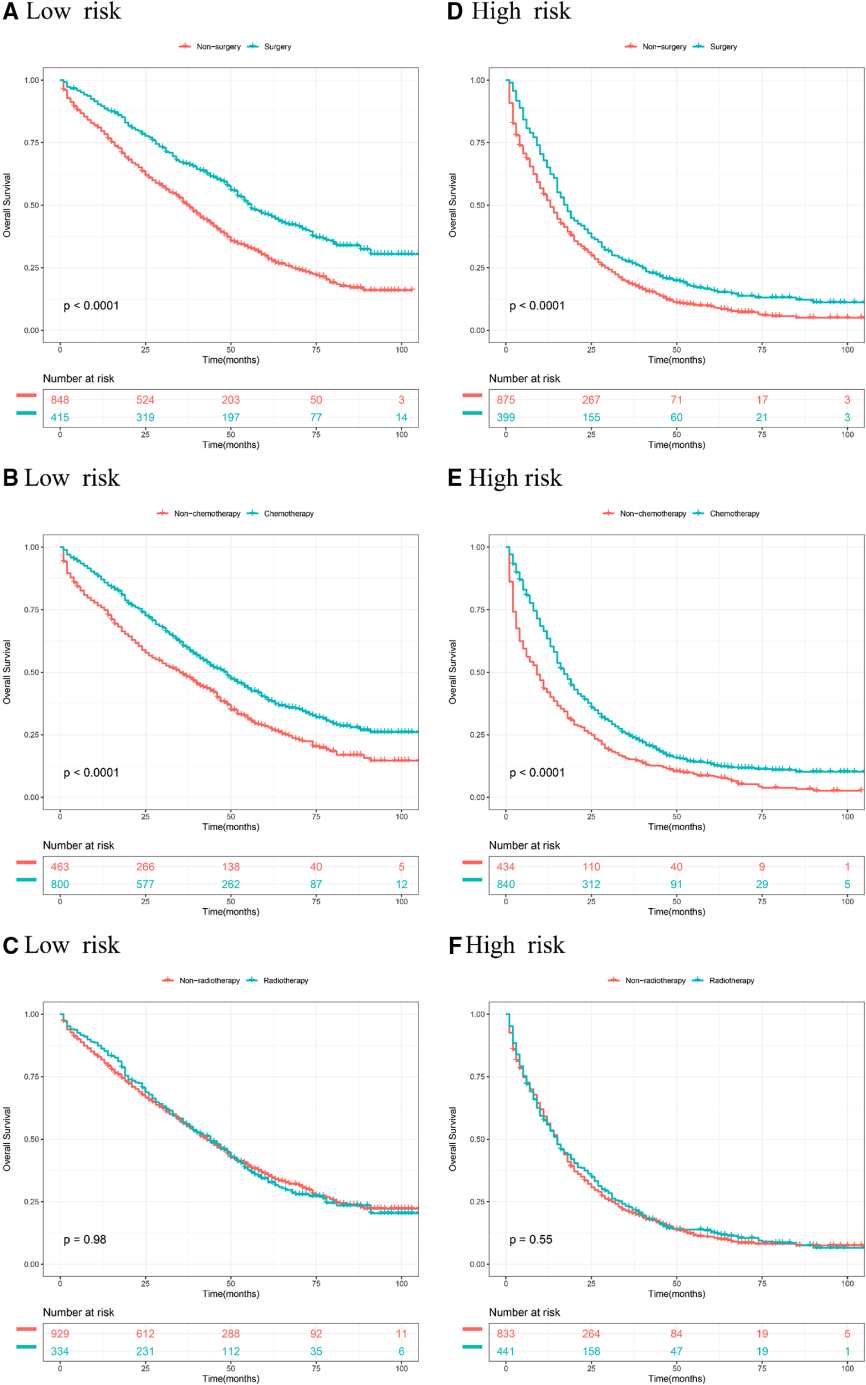
Kaplan–meier curves of different treatments for risk stratification in terms of OS. Kaplan–Meier curves of primary surgery in the low-risk group **(A)** and high-risk group **(B)**; Kaplan–Meier curves of chemotherapy in the low-risk group **(C)** and the high-risk group **(D)**; Kaplan–Meier curves of radiotherapy in the low-risk group **(E)** and the high-risk group **(F)**.

**Figure 7:**
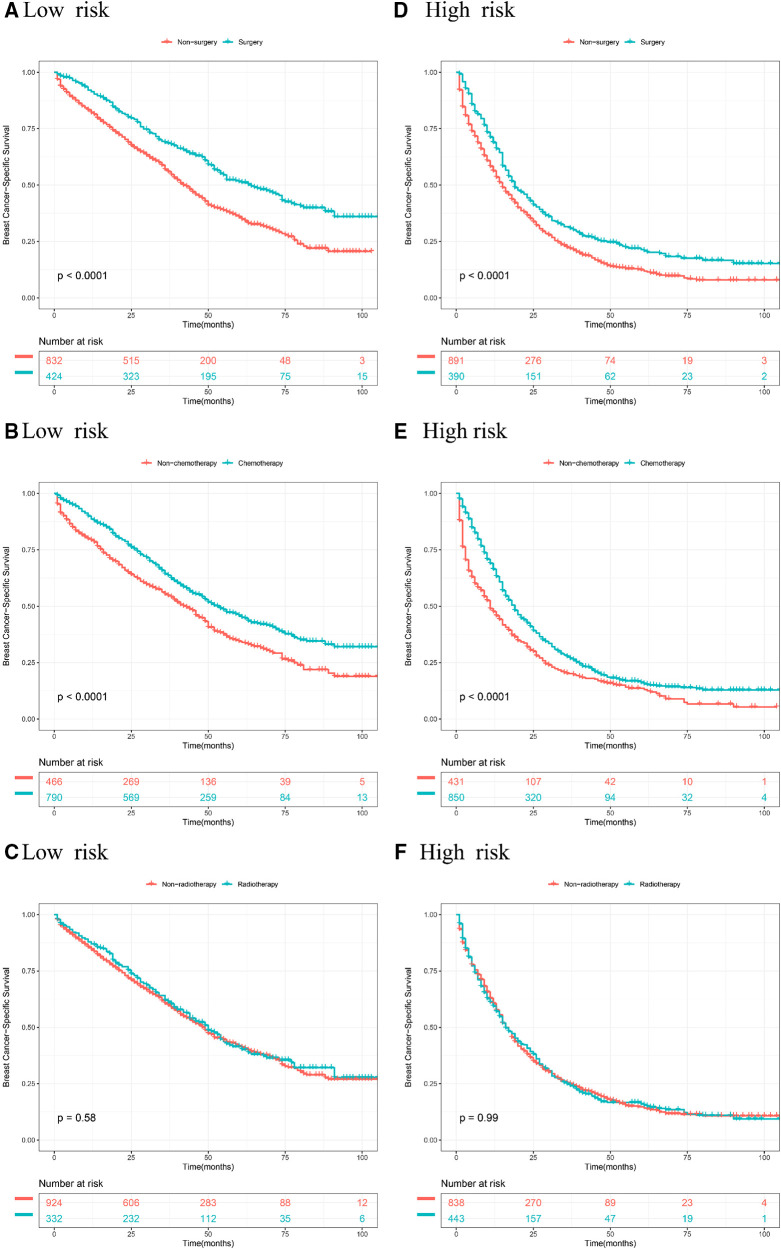
Kaplan–Meier curves of different treatments for risk stratification in terms of BCSS. Kaplan–Meier curves of primary surgery in the low-risk group **(A)** and high-risk group **(B)**; Kaplan–Meier curves of chemotherapy in the low-risk group **(C)** and the high-risk group **(D)**; Kaplan–Meier curves of radiotherapy in the low-risk group **(E)** and the high-risk group **(F)**.

According to stratified risk groups, we further investigated the survival benefit of primary site surgery, chemotherapy, and radiotherapy for BCLM patients. As illustrated in Figures 6A,D, primary site surgery remarkably prolonged OS of BCLM patients in both the low-risk (*P* < 0.0001) and high-risk groups (*P* < 0.0001). Furthermore, chemotherapy had a favorable effect on the OS of BCLM patients in both the low-risk (*P* < 0.0001) and high-risk groups (*P* < 0.0001) (Figures 6B,E). However, radiotherapy neither improved the OS of BCLM patients in the low-risk group (*P* = 0.98) nor improved the OS of those in the high-risk group (*P* = 0.55) (Figures 6C,F). The same outcomes could be observed for BCSS of BCLM patients as shown in Figure 7. The outcomes above showed that primary site surgery and chemotherapy are beneficial to BCLM patients, whether they are at low-risk or high-risk.

## Discussion

It is universally known that diverse clinicopathological parameters and molecular characteristics are closely related to clinical outcomes in BCLM patients. For example, a retrospective study reported that the prognosis of BCLM patients is dissatisfactory, with an 11-month median survival time in TNBC, and better outcomes of 31 months in HR+/HER2 +  (4). BCLM patients who suffer the additional metastatic disease at distant sites (brain, liver, bone) obtain worse survival results compared to patients without distant metastases (8). For the complexity of the multivariate prognostic factors affecting the survival of BCLM patients, it was difficult to estimate the survival outcomes for BCLM patients. Therefore, we developed two prognostic nomograms to predict OS and BCSS for BCLM patients.

In the present study, age, marital status, race, laterality, grade, AJCC T stage, subtype, bone metastasis, brain metastasis, liver metastasis, surgery, and chemotherapy were found to be independent predictors of OS and BCSS. Chen S et al. have reported that age, race, marital status, pathological grade, molecular subtype, and extrapulmonary metastatic sites were survival risk factors for BCLM (5). In BCLM, age, black race, HR−/HER2 + subtype, triple-negative subtype, and higher grade had an adverse influence on the long-term prognosis of patients, while HR+/HER2 + subtype and marital status showed a favorable effect on the long-term survival of patients (4). These results were generally consistent with our reports. Additionally, we found that laterality is also a survival predictor for BCLM patients, and breast cancer on the left side has a better prognosis than breast cancer on the right side, which is not shown in other studies. The reason for this may be selection bias, and more studies and further prospective randomized trials with rigorous inclusion criteria are eagerly awaited to verify our results. In addition, the T stage was also associated with the prognosis of BCLM, and a lower T stage implied better survival. Furthermore, other factors mentioned above, primary site surgery and chemotherapy were also identified as significant predictors of prognosis.

Two nomograms were established to visualize the predictive survival of BCLM patients based on the results of multivariate Cox analysis. The nomograms in the present study could accurately estimate the prognosis of BCLM patients, which is helpful to the clinical management of patients. For the purpose of better understanding the use of the nomograms, we took a patient with BCLM as an example. A 50-year-old woman, married, white, right side of breast cancer, grade IV, AJCC T4, HR-/HER-, with lung metastases from breast cancer, and no metastases beyond the lung, received surgery and chemotherapy, the patient had approximately 67%, 45%, and 28% of 1-, 2-, and 3-year overall survival probabilities, respectively. Traditionally, the main treatment for metastatic breast cancer is normally palliative care and supportive care, which aims at maintaining the quality of life and relieving symptoms. An accurate survival estimation can assist clinicians and patients in making the most appropriate treatment plan, and was conducive to the rational utilization and allocation of medical resources. If the predicted survival rate is good, we can choose a more aggressive treatment strategy. If the predicted survival rate is poor, negative treatment methods such as palliative care and supportive care are more suitable for the patients, so as to avoid the side effects caused by aggressive treatment and improve the quality of life. Predicting the survival risk of BCLM patients can facilitate individualized treatment regimens, which is of great significance for improving the prognosis and quality of life for BCLM patients.

We used multiple methods to verify the clinical efficacy of the constructed nomograms. The predictive performance of the nomograms was evaluated by discrimination and calibration internally and externally. The C-index was approximately 0.7, suggesting a good discrimination ability of the nomograms. The AUC values of 0.7 to 0.8 indicated that our nomograms showed great predictive ability for the prognosis of BCLM patients. The calibration curves showed excellent consistency between the actual observations and the predicted outcomes in predicting OS and BCSS, which guaranteed the reliability of the established nomograms. We also stratified the prognostic risk of BCLM patients based on nomograms. The significant difference in Kaplan–Meier curves among the low-risk and the high-risk groups confirmed the excellent predictive ability of the nomograms.

In our research, primary site surgery and chemotherapy could remarkably prolong OS and BCSS of BCLM patients no matter whether the patients were at low-risk or high-risk. Currently, chemotherapy, targeted therapy, and endocrine therapy are beneficial to the long-term survival of metastatic breast cancer and are the first-line treatment strategies for advanced breast cancer. But the role of primary site surgery (breast resection) in metastatic breast cancer is still controversial. For stage IV breast cancer, resection of the primary tumor can reduce tumor burden and control cancer-related symptoms. Conversely, it has also been reported that primary site surgery may accelerate the emergence of distant metastasis by inducing angiogenesis and proliferation of distant dormant micrometastases (9). In terms of existing evidence, some studies showed that breast cancer patients with bone metastasis alone can benefit from resection of the primary tumor, while patients with visceral metastasis do not (10–12). However, another study showed that surgery is related to better OS in breast cancer patients with single metastasis to the liver, lung, or brain (13). The NCCN guidelines for breast cancer suggest that surgery at the primary site is not recommended except for patients who can benefit from initial systemic therapy (14). Radiotherapy, as a local treatment, is often used as adjuvant therapy for breast cancer receiving breast-conserving surgery. Radiotherapy also has been a palliative treatment strategy that aims to control tumor progression and suppress tumor-related symptoms for cancer patients with metastatic diseases. Radiotherapy had improvement in locoregional recurrence, however, this does not translate into an advantage in the overall survival of early-stage breast cancer patients (15, 16). Few high-evidence studies like randomized controlled trials were conducted to investigate the effect of radiotherapy among *de novo* stage IV breast cancer patients so far. Our results showed that radiotherapy did not improve the survival outcomes of BCLM patients. But as an effective strategy in controlling local lesions, radiotherapy is often used in combination with drug therapy for advanced breast cancer. Our results could provide some basis for the treatment choice of patients with BCLM to some extent.

Inevitably, some limitations were in this research. First, there is no data on the different options of the systemic treatment used. Endocrine therapy and targeted therapy play vital roles in the treatment of metastatic or advanced breast cancer, but the information was not recorded in the SEER database, leading to the deviation of patient survival prediction to some extent. Second, information about lung metastases was absent, such as the data on the type of metastatic lesions to the lungs (single, multiple). A large number of retrospective studies have presented obvious benefits for BCLM patients who undergo pulmonary metastasectomy (17–20). The number of lung metastasis influences the choice of the further procedure because a single lesion to the lung was possibly to select surgical excision, and the lack of relevant information may affect the accuracy of the model in predicting survival. Third, other metastatic sites that may affect the prognosis of metastatic breast cancer, such as the peritoneum, other internal organs, or skin, were not collected in this study. Fourth, we do not take the general condition of patients into account owing to the inherent biases in the SEER database (21), which often affects the therapeutic possibilities. Finally, although our models showed excellent predictive performance, they had not been validated in other centers or databases.

In conclusion, age, marital status, race, laterality, grade, AJCC T stage, subtype, bone metastasis, brain metastasis, liver metastasis, surgery, and chemotherapy were identified as independent prognostic indicators for BCLM. The first prognostic nomogram created for BCLM can excellently predict individual survival and assist clinicians in optimizing individualized treatment strategies for BCLM patients.

## Data Availability

Publicly available datasets were analyzed in this study. This data can be found here: https://seer.cancer.gov/.
