# Supplementary material for: An effective tool for predicting survival in breast cancer patients with de novo lung metastasis: Nomograms constructed based on SEER
Source: Front Surg. 2023 Jan 6;9:939132. doi: 10.3389/fsurg.2022.939132 (PMC9852616; doi:10.3389/fsurg.2022.939132)
Supplement: Supplementary file 1 [file Table1.docx]

**SUPPLEMENTARY TABLE 1** Univariate COX regression analysis based on all variables for OS and BCSS.

| **Variables** | **OS** | |  |  | **BCSS** | |  |
| --- | --- | --- | --- | --- | --- | --- | --- |
|  | **HR (95%CI)** | **P-value** |  |  | **HR (95%CI)** | **P-value** |  |
| **Sex** |  |  |  |  |  |  |  |
| Female | Reference |  |  |  | Reference |  |  |
| Male | 0.723(0.474-1.103) | 0.1320 |  |  | 0.637(0.394-1.028) | 0.0648 |  |
| **Age** |  |  |  |  |  |  |  |
| <40 | Reference |  |  |  | Reference |  |  |
| 40-59 | 1.184(0.926-1.514) | 0.1771 |  |  | 1.210(0.935-1.567) | 0.1474 |  |
| 60-79 | 1.312(1.0290-1.674) | 0.0285 |  |  | 1.247(0.965-1.612) | 0.0913 |  |
| 80+ | 2.343(1.784-3.077) | 0.0000 |  |  | 2.094(1.565-2.803) | 0.0000 |  |
| **Marital status** |  |  |  |  |  |  |  |
| Married | Reference |  |  |  | Reference |  |  |
| Unmarried | 1.457(1.306-1.625) | 0.0000 |  |  | 1.410(1.255-1.584) | 0.0000 |  |
| **Race** |  |  |  |  |  |  |  |
| White | Reference |  |  |  | Reference |  |  |
| Black | 1.290(1.128-1.476) | 0.0002 |  |  | 1.289(1.117-1.488) | 0.0005 |  |
| Other | 0.843(0.688-1.033) | 0.1000 |  |  | 0.836(0.673-1.039) | 0.1061 |  |
| **Site** |  |  |  |  |  |  |  |
| Inner | Reference |  |  |  | Reference |  |  |
| Outer | 0.905(0.749-1.094) | 0.3040 |  |  | 0.911(0.743-1.117) | 0.3700 |  |
| Other | 1.0667(0.897-1.269) | 0.4660 |  |  | 1.099(0.912-1.325) | 0.3190 |  |
| **Laterality** |  |  |  |  |  |  |  |
| Left | Reference |  |  |  | Reference |  |  |
| Right | 1.152(1.035-1.282) | 0.0098 |  |  | 1.146( 1.022-1.285) | 0.0197 |  |
| **Grade** |  |  |  |  |  |  |  |
| I-II | Reference |  |  |  | Reference |  |  |
| III-IV | 1.413(1.266-1.577) | 0.0000 |  |  | 1.531(1.360-1.724) | 0.0000 |  |
| **AJCC_T** |  |  |  |  |  |  |  |
| T1-2 | Reference |  |  |  | Reference |  |  |
| T3-4 | 1.370(1.229-1.542) | 0.0000 |  |  | 1.441(1.276-1.628) | 0.0000 |  |
| **AJCC_N** |  |  |  |  |  |  |  |
| N0 | Reference |  |  |  | Reference |  |  |
| N1-3 | 0.977(0.856-1.116) | 0.7330 |  |  | 1.049(0.908-1.212) | 0.5130 |  |
| **Subtype** |  |  |  |  |  |  |  |
| HR+/HER2- | Reference |  |  |  | Reference |  |  |
| HR+/HER2+ | 0.749(0.643-0.874) | 0.0002 |  |  | 0.779(0.661-.917) | 0.0027 |  |
| HR-/HER2+ | 1.086(0.901-1.310) | 0.3867 |  |  | 1.075(0.877-.318) | 0.4843 |  |
| HR-/HER2- | 2.342 (2.044-2.682) | 0.0000 |  |  | 2.553(2.213-.945) | 0.0000 |  |
| **bone** |  |  |  |  |  |  |  |
| No | Reference |  |  |  | Reference |  |  |
| Yes | 1.225(1.099-1.364) | 0.0002 |  |  | 1.195(1.065-1.341) | 0.0024 |  |
| **brain** |  |  |  |  |  |  |  |
| No | Reference |  |  |  | Reference |  |  |
| Yes | 2.137(1.806-2.528) | 0.0000 |  |  | 2.186(1.830-2.611) | 0.0000 |  |
| **liver** |  |  |  |  |  |  |  |
| No | Reference |  |  |  | Reference |  |  |
| Yes | 1.573(1.401-1.767) | 0.0000 |  |  | 1.698(1.502-1.918) | 0.0000 |  |
| **Surgery** |  |  |  |  |  |  |  |
| No | Reference |  |  |  | Reference |  |  |
| Yes | 0.724(0.644-0.813) | 0.0000 |  |  | 0.721(0.636-.816) | 0.0000 |  |
| **Chemotherapy** | |  |  |  |  |  |  |
| No/Unknown | Reference |  |  |  | Reference |  |  |
| Yes | 0.707(0.633-0.790) | 0.0000 |  |  | 0.756(0.671-.852) | 0.0000 |  |
| **Radiation** |  |  |  |  |  |  |  |
| No/Unknown | Reference |  |  |  | Reference |  |  |
| Yes | 1.115(0.994-1.252) | 0.0637 |  |  | 1.118(0.989-1.265) | 0.0750 |  |

For marital status, unmarried consists of unmarried, single, divorced, separated, and widowed;

For race, ‘other’ includes American Indian, AK Native, Asian, and Pacific Islander;

For grade, Grade Ⅰ means well-differentiated, grade Ⅱ means moderately differentiated, grade III means poorly differentiated, Grade Ⅳ means undifferentiated or anaplastic
