# Supplementary material for: An effective tool for predicting survival in breast cancer patients with de novo lung metastasis: Nomograms constructed based on SEER
Source: Front Surg. 2023 Jan 6;9:939132. doi: 10.3389/fsurg.2022.939132 (PMC9852616; doi:10.3389/fsurg.2022.939132)
Supplement: Supplementary file 2 [file Table2.docx]

**SUPPLEMENTARY TABLE 2** C-index for internal and external validation.

| Model | C-index (95% CI) | |
| --- | --- | --- |
|  | Internal validation | External validation |
| OS | Concordance= 0.701 (0.687-0.715) | 0.699 (0.677-0.721) |
| BCSS | Concordance= 0.708 (0.692-0.724) | 0.697 (0.673-0.721) |
