# Supplementary material for: An effective tool for predicting survival in breast cancer patients with de novo lung metastasis: Nomograms constructed based on SEER
Source: Front Surg. 2023 Jan 6;9:939132. doi: 10.3389/fsurg.2022.939132 (PMC9852616; doi:10.3389/fsurg.2022.939132)
Supplement: Supplementary file 3 [file Table3.docx]

**TABLE 1** Demographics and clinicopathologic characteristics of the cohort with BCLM.

| **Variables** | Overall(N=2537) | Training cohort(N=1777) | Validation cohort(N=760) | P-value |
| --- | --- | --- | --- | --- |
| **Sex** |  |  |  | 0.862 |
| Female | 2497 (98.4%) | 1748 (98.4%) | 749 (98.6%) |  |
| Male | 40 (1.6%) | 29 (1.6%) | 11 (1.4%) |  |
| **Age** |  |  |  | 0.708 |
| <40 | 166 (6.5%) | 113 (6.4%) | 53 (7.0%) |  |
| 40-59 | 956 (37.7%) | 682 (38.4%) | 274 (36.1%) |  |
| 60-79 | 1130 (44.5%) | 784 (44.1%) | 346 (45.5%) |  |
| 80+ | 285 (11.2%) | 198 (11.1%) | 87 (11.4%) |  |
| **Marital status** |  |  |  | 0.573 |
| Married | 1116 (44.0%) | 788 (44.3%) | 328 (43.2%) |  |
| Unmarried | 1421 (56.0%) | 989 (55.7%) | 432 (56.8%) |  |
| **Race** |  |  |  |  |
| White | 1833 (72.3%) | 1294 (72.8%) | 539 (70.9%) | 0.6 |
| Black | 484 (19.1%) | 334 (18.8%) | 150 (19.7%) |  |
| Other | 220 (8.7%) | 149 (8.4%) | 71 (9.3%) |  |
| **Site** |  |  |  | 0.676 |
| Inner | 277 (10.9%) | 200 (11.3%) | 77 (10.1%) |  |
| Outer | 733 (28.9%) | 515 (29.0%) | 218 (28.7%) |  |
| Other | 1527 (60.2%) | 1062 (59.8%) | 465 (61.2%) |  |
| **Laterality** |  |  |  | 0.516 |
| Left | 1272 (50.1%) | 883 (49.7%) | 389 (51.2%) |  |
| Right | 1265 (49.9%) | 894 (50.3%) | 371 (48.8%) |  |
| **Grade** |  |  |  | 0.511 |
| I-II | 1076 (42.4%) | 746 (42.0%) | 330 (43.4%) |  |
| III-IV | 1461 (57.6%) | 1031 (58.0%) | 430 (56.6%) |  |
| **AJCC_T** |  |  |  | 0.655 |
| T1-2 | 957 (37.7%) | 665 (37.4%) | 292 (38.4%) |  |
| T3-4 | 1580 (62.3%) | 1112 (62.6%) | 468 (61.6%) |  |
| **AJCC_N** |  |  |  | 0.789 |
| N0 | 523 (20.6%) | 369 (20.8%) | 154 (20.3%) |  |
| N1-3 | 2014 (79.4%) | 1408 (79.2%) | 606 (79.7%) |  |
| **Subtype** |  |  |  | 0.248 |
| HR+/HER2- | 1293 (51.0%) | 897 (50.5%) | 396 (52.1%) |  |
| HR+/HER2+ | 471 (18.6%) | 346 (19.5%) | 125 (16.4%) |  |
| HR-/HER2+ | 276 (10.9%) | 185 (10.4%) | 91 (12.0%) |  |
| HR-/HER2- | 497 (19.6%) | 349 (19.6%) | 148 (19.5%) |  |
| **bone** |  |  |  | 0.165 |
| No | 1215 (47.9%) | 835 (47.0%) | 380 (50.0%) |  |
| Yes | 1322 (52.1%) | 942 (53.0%) | 380 (50.0%) |  |
| **brain** |  |  |  | 0.199 |
| No | 2307 (90.9%) | 1607 (90.4%) | 700 (92.1%) |  |
| Yes | 230 (9.1%) | 170 (9.6%) | 60 (7.9%) |  |
| **liver** |  |  |  | 0.244 |
| No | 1842 (72.6%) | 1278 (71.9%) | 564 (74.2%) |  |
| Yes | 695 (27.4%) | 499 (28.1%) | 196 (25.8%) |  |
| **Surgery** |  |  |  | 0.378 |
| No | 1723 (67.9%) | 1197 (67.4%) | 526 (69.2%) |  |
| Yes | 814 (32.1%) | 580 (32.6%) | 234 (30.8%) |  |
| **Chemotherapy** |  |  |  | 0.174 |
| No/Unknown | 897 (35.4%) | 613 (34.5%) | 284 (37.4%) |  |
| Yes | 1640 (64.6%) | 1164 (65.5%) | 476 (62.6%) |  |
| **Radiation** |  |  |  | 0.371 |
| No/Unknown | 1762 (69.5%) | 1244 (70.0%) | 518 (68.2%) |  |
| Yes | 775 (30.5%) | 533 (30.0%) | 242 (31.8%) |  |

For marital status, unmarried consists of unmarried, single, divorced, separated, and widowed;

For race, ‘other’ includes American Indian, AK Native, Asian, and Pacific Islander;

For grade, Grade Ⅰ means well-differentiated, grade Ⅱ means moderately differentiated, grade III means poorly differentiated, Grade Ⅳ means undifferentiated or anaplastic
