# Supplementary material for: An effective tool for predicting survival in breast cancer patients with de novo lung metastasis: Nomograms constructed based on SEER
Source: Front Surg. 2023 Jan 6;9:939132. doi: 10.3389/fsurg.2022.939132 (PMC9852616; doi:10.3389/fsurg.2022.939132)
Supplement: Supplementary file 4 [file Table4.docx]

**TABLE 2** Multivariate COX regression analysis for overall survival (OS) and breast cancer-specific survival (BCSS) of BCLM patients in the training cohort.

| **Variables** | **OS** | | |  | **BCSS** | | |
| --- | --- | --- | --- | --- | --- | --- | --- |
|  | **HR (95%CI)** | **P-value** | **Points** |  | **HR (95%CI)** | **P-value** | **Points** |
| **Sex** |  |  |  |  |  |  |  |
| Female | - | - | - |  | - | - | - |
| Male | - | - | - |  | - | - | - |
| **Age** |  |  |  |  |  |  |  |
| <40 | Reference |  | 0 |  | Reference |  | 0 |
| 40-59 | 1.288(1.005-1.651) | 0.0453 | 19 |  | 1.322(1.019-1.715) | 0.0358 | 20 |
| 60-79 | 1.480(1.155-1.897) | 0.0020 | 30 |  | 1.427(1.099-1.854) | 0.0077 | 26 |
| 80+ | 2.458(1.844-3.277) | 0.0000 | 68 |  | 2.264(1.666-3.078) | 0.0000 | 60 |
| **Marital status** |  |  |  |  |  |  |  |
| Married | Reference |  | 0 |  | Reference |  | 0 |
| Unmarried | 1.319(1.177-1.478) | 0.0000 | 21 |  | 1.2991.151-1.466) | 0.0000 | 19 |
| **Race** |  |  |  |  |  |  |  |
| White | Reference |  | 14 |  | Reference |  | 15 |
| Black | 1.228(1.065-1.415) | 0.0047 | 30 |  | 1.184(1.017-1.377) | 0.0293 | 27 |
| Other | 0.833(0.678-1.023) | 0.0819 | 0 |  | 0.814(0.653-1.014) | 0.0660 | 0 |
| **Site** |  |  |  |  |  |  |  |
| Inner | - | - | - |  | - | - | - |
| Outer | - | - | - |  | - | - | - |
| Other | - | - | - |  | - | - | - |
| **Laterality** |  |  |  |  |  |  |  |
| Left | Reference |  | 0 |  | Reference |  | 0 |
| Right | 1.160(1.041-1.293) | 0.0074 | 11 |  | 1.163(1.036-1.306) | 0.0104 | 11 |
| **Grade** |  |  |  |  |  |  |  |
| I-II | Reference |  | 0 |  | Reference |  | 0 |
| III-IV | 1.402(1.236-1.590) | 0.0000 | 26 |  | 1.493(1.304-1.709) | 0.0000 | 20 |
| **AJCC_T** |  |  |  |  |  |  |  |
| T1-2 | Reference |  | 0 |  | Reference |  | 0 |
| T3-4 | 1.307(1.164-1.468) | 0.0000 | 20 |  | 1.354(1.196-1.534) | 0.0000 | 22 |
| **AJCC_N** |  |  |  |  |  |  |  |
| N0 | - | - | - |  | - | - | - |
| N1-3 | - | - | - |  | - | - | - |
| **Subtype** |  |  |  |  |  |  |  |
| HR+/HER2- | Reference |  | 18 |  | Reference |  | 18 |
| HR+/HER2+ | 0.789(0.669-0.930) | 0.0048 | 0 |  | 0.777(0.652-0.926) | 0.0047 | 0 |
| HR-/HER2+ | 1.207(0.982-1.485) | 0.0738 | 32 |  | 1.102(0.881-1.377) | 0.3950 | 26 |
| HR-/HER2- | 2.937(2.492-3.462) | 0.0000 | 100 |  | 3.051(2.566-3.629) | 0.0000 | 100 |
| **bone** |  |  |  |  |  |  |  |
| No | Reference |  | 0 |  | Reference |  | 0 |
| Yes | 1.355(1.198-1.532) | 0.0000 | 23 |  | 1.3206(1.1583-1.5057) | 0.0000 | 20 |
| **brain** |  |  |  |  |  |  |  |
| No | Reference |  | 0 |  | Reference |  | 0 |
| Yes | 1.926(1.618-2.294) | 0.0000 | 50 |  | 1.9160(1.593-2.305) | 0.0000 | 48 |
| **liver** |  |  |  |  |  |  |  |
| No | Reference |  | 0 |  | Reference |  | 0 |
| Yes | 1.644(1.451-1.863) | 0.0000 | 38 |  | 1.778(1.559-2.028) | 0.0000 | 42 |
| **Surgery** |  |  |  |  |  |  |  |
| No | Reference |  | 20 |  | Reference |  | 21 |
| Yes | 0.770(0.680-0.872) | 0.0000 | 0 |  | 0.747(0.654-0.853) | 0.0000 | 0 |
| **Chemotherapy** | |  |  |  |  |  |  |
| No/Unknown | Reference |  | 35 |  | Reference |  | 32 |
| Yes | 0.633(0.556-0.721) | 0.0000 | 0 |  | 0.648(0.563-0.745) | 0.0000 | 0 |
| **Radiation** |  |  |  |  |  |  |  |
| No/Unknown | - | - | - |  | - | - | - |
| Yes | - | - | - |  | - | - | - |
